# Advance Care Planning in Dementia: Do Family Carers Know the Treatment Preferences of People with Early Dementia?

**DOI:** 10.1371/journal.pone.0159056

**Published:** 2016-07-13

**Authors:** Karen Harrison Dening, Michael King, Louise Jones, Victoria Vickestaff, Elizabeth L. Sampson

**Affiliations:** 1 Dementia UK, Second floor, Resource for London, London, United Kingdom; 2 Marie Curie Palliative Care Research Department, UCL Division of Psychiatry, 6th Floor, Maple House, London, United Kingdom; 3 Division of Psychiatry, Faculty of Brain Sciences, UCL, Maple House, London, United Kingdom; ISPO, ITALY

## Abstract

**Background and Aims:**

When a person with dementia (PWD) has lost the ability to make treatment decisions, clinicians often rely on family carers to know and articulate these preferences with assumed accuracy. This study used the Life Support Preferences Questionnaire (LSPQ) to explore whether family carers’ choices show agreement with the end of life care preferences of the person with dementia for whom they care and what factors influence this.

**Methods:**

A cross-sectional study interviewing 60 dyads (a person with early dementia and preserved capacity and their family carer) each completing a modified LSPQ. We assessed how closely carers’ choices resembled the PWD’s preferences for treatment in three proposed health states: the here and now; severe stroke with coma; terminal cancer. Agreement between the PWD and their family carer responses was assessed using Kappa and Prevalence-Adjusted Bias-Adjusted Kappa (PABAK) statistics. We examined whether carer burden and distress, and relationship quality, influenced agreement.

**Results:**

In interviews PWD were able to indicate their treatment preferences across all three scenarios. In the *here-and-now* most wanted antibiotics (98%), fewer cardio-pulmonary resuscitation (CPR) (50%) and tube feeding (47%). In *severe stroke and coma* antibiotics remained the more preferred treatment (88%), followed by CPR (57%) and tube feeding (30%). In *advanced cancer* PWD expressed lower preferences for all treatments (antibiotics 68%; CPR 50%; tube feeding 37%). Carers’ choices were similar to the PWDs’ preferences in the *here-and-now* (71% (***k*** = 0.03; PABAK = 0.4) with less agreement for future hypothetical health states. In *severe stroke and coma* carers tended wrongly to suggest that the PWD preferred more intervention (antibiotic, 67%; ***k*** = -0.022; PABAK = -0.60; CPR, 73%; ***k*** = 0.20; PABAK = -0.20, tube feeding, 66%; ***k*** = 0.25; PABAK = -0.12). In *advanced cancer* the agreement between PWD and carers was low (antibiotics; ***k*** = -0.03; PABAK = -0.52; CPR, ***k*** = -0.07; PABAK = -0.45; tube feeding; ***k*** = 0.20; PABAK = -0.22). However, both PWD and carers showed marked uncertainty about their preferences for end of life treatment choices. Relationship quality, carer distress and burden had no influence on agreement.

**Conclusions:**

This study is the first to have used the LSPQ with PWD in the UK to consider treatment options in hypothetical illness scenarios. Key finding are that family carers had a low to moderate agreement with PWD on preferences for end of life treatment. This underscores how planning for care at the end of life is beset with uncertainty, even when the carer and PWD perceive the care-giving/receiving relationship is good. Families affected by dementia may benefit from early and ongoing practical and emotional support to prepare for potential changes and aid decision making in the context of the realities of care towards the end of life.

## Introduction

### An ageing population and dementia

There are an estimated 835,000 people currently living with dementia in the UK which will rise to over two million by 2050 [1]. Dementia is a degenerative disease and thus a life-limiting condition. Failure to recognise dementia as a terminal illness and that it is often accompanied by multi morbidity has, until recently, led to neglect in addressing end of life challenges for people with dementia (PWD) and their carers [2].

### Planning end of life care in dementia

Interest in palliative and end of life care for people with dementia is increasing [3,4]. The UK, the End of Life Care Strategy [5], English National Dementia Strategy [6] and Prime Ministers Challenge [7] have all called for improvement in care towards the end of life in dementia by promoting ‘advance care planning’. It has been suggested that *all* people should be encouraged to identify their needs, priorities and preferences for end of life care [5]. This may be challenging for people with cognitive impairment and in those with reduced capacity to express their preferences.

### Dementia and decision making

Autonomy in decision making depends upon consciousness of our past and future thoughts and actions in the same way as we are conscious of our present thoughts and actions [8]. However, as dementia progresses, in particular the ability to consider future thoughts and actions become compromised and this affects the capacity to make decisions [9]. Capacity, in the context of decision making, refers to the ability to consider and weigh up information relevant to a situation, retain it and communicate that decision [10]. In dementia decisions vary in complexity and importance, ranging for example from decisions to be made about a health crisis to those about day to day needs. Older people often trust loved ones to make healthcare decisions on their behalf [11] and want those decisions to be in keeping with their own wishes and preferences [12]. Family carers are assumed to know what these wishes and preferences would have been had the person with dementia not lost capacity [13] and professionals often rely on family members to predict and articulate these preferences with assumed accuracy [14].

### Accuracy in predicting the wishes and preferences of the person with dementia

Making decisions about end of life care and treatment on behalf of a family member may be difficult, for example considering whether to withhold treatment, choosing between treatment options and taking note of the context of the decision, for example, in a crisis as compared to states of chronic ill-health [15,16]. Family carers’ patterns of decision-making differ according to their previous experiences of end of life care [17], education, perceived carer burden [18], psychological distress and cultural background [19]. Not surprisingly, carers often find making health-related decisions for the person they care for stressful [20]. Decisions concerning end of life care are amongst the most difficult [21]. Families who are in conflict or with poor inter-relational dynamics may be more likely to opt for active treatment [22,23] rather than palliative care [23]. However, even in the absence of such conflict, when a family is in doubt or uncertain as to what to decide, they may err on the side of caution and elect for life-sustaining treatment for the person with dementia.

### Aims and objectives

In this study we aimed to increase understanding of the treatment and care preferences of PWD and particularly whether family carers’ choices are in agreement.

Our specific objectives were: a) to use a modified version of the Life Support Preferences Questionnaire (LSPQ) [24] to explore the choices PWD make for end of life care, b) to examine how accurately family carers of a PWD agree with their wishes and preferences for end of life care, c) to examine factors that might influence this agreement and levels of uncertainty in choices for treatment in both the PWD and the family carer.

## Methods

### Design and Study population

A cross sectional interview study of dyads of PWD and their family carer or close friend.

### Recruitment

Dyads were identified by psychiatrists and registered nurses in memory clinics, community mental health teams or coordinators of dementia research registers in four different sites in England: 1) Barnet Enfield & Haringey Mental Health Trust, 2) Cambridge and Peterborough Foundation Trust, 3) West London Mental Health Trust and 4) Leicestershire Partnership Trust.

Inclusion criteria for PWD were a clinical diagnosis of any type of dementia as categorised in ICD-10 [25], a Mini Mental State Examination score [26] ≥ 20 and the mental capacity to consent to and participate in the interview. Carers were included if identified as next of kin or key decision maker for the PWD. Dyads were excluded if either the PWD or carer did not consent to take part and if either was unable to communicate sufficiently in English, as no funding was available for use of interpreters.

### Procedure

Potential participants were identified by clinicians in each of the four sites. They assessed the capacity of both the PWD and carer who expressed interest in involvement. The researcher then made contact with the nominated contact person of each dyad, usually the carer, to arrange the interview. Capacity of the PWD was reassessed by the researcher (KHD) prior to commencement of the interview. Interviews were held in the participants’ choice of location.

The aims of the study were explained to the both the PWD and their carer. Written consent from both the PWD and carer was then obtained. Each participant was interviewed separately; the PWD first and carer second. Interviews lasted approximately one hour and were carried out by KHD between April 2012 and February 2014. If either the PWD or their carer became distressed at any time during the research process, they were offered the support of Admiral Nursing DIRECT and if necessary the interview was terminated.

### Socio-demographic data

We recorded the socio-demographic characteristics of the PWD and carer (age, gender, ethnicity, education, employment, and living situation), as well as the relationship between the carer/friend and PWD.

### Measures: people with dementia

We used two instruments:

1. Quality of Carer Patient Relationship (QCPR) [27]. This is a 14-item scale that examines agreement on quality of the relationship between a caregiver and a care recipient. Responses are given on a 5-point Likert scale, ranging from ‘totally disagree’ to ‘totally agree’, giving a range of scores from 14 to 70, with higher values representing better relationships.
2. A modified version of the Life Support Preferences Questionnaire (LSPQ) [24]. The LSPQ questionnaire is an American tool using nine health state scenarios to support the development of advance directives. As our aim was to explore how well carers’ agreed with the treatment preferences of the PWD, we modified the LSPQ to three scenarios for participants to consider. One scenario featured the prospect of developing dementia, and as our target population already had a diagnosis of dementia, we described our first scenario as, ‘*as you are today*, *with mild memory problems’*. The two other scenarios we selected were stroke with coma and advanced cancer as most people understand or have had experience of these conditions amongst family and friends (see Box 1).

**Box 1. Life Support Preference Questionnaire Health states**

**SCENARIO 1**

**Imagine** you are in your current health, in other words, the way you are feeling now.

**SCENARIO 2**

**Imagine** you have suffered a severe stroke and have been in a coma for six weeks.

In the opinion of your doctor, you have no chance of regaining awareness or the ability to think, reason, and remember.

Your current physical condition is stable, but will slowly decline over time. You rely on others for help with feeding, bathing, dressing, and toileting. You may live in this condition for several years.

**SCENARIO 3**

**Imagine** you have advanced cancer and it has spread to other areas. You are tired and weak, requiring some help with household chores. **Imagine** you have a pain that requires the constant use of medication. In the opinion of your doctor, you have no chance of recovery. Your doctor estimates that you have about six months to live.

People with dementia were asked to consider themselves in each scenario and indicate their preference for receiving three discrete life-sustaining medical treatments in each of the three scenarios using a five point Likert Scale (‘definitely would want’, ‘probably would want’, ‘unsure’, ‘probably would not want’ and ‘definitely would not want’). The treatments were antibiotics, cardio-pulmonary resuscitation and tube feeding. Each treatment was explained to participants as often as necessary and a ‘prompt card’ (see Box 2) was used when needed.

**Box 2. Life Support Preference Questionnaire Treatment choices**

**1. Antibiotics**

Doctors use these medicines to treat serious infections (e.g., pneumonia). Without antibiotics, serious infections can cause life threatening complications or death.

**2. Cardiopulmonary Resuscitation (CPR)**

Doctors use cardiopulmonary resuscitation, or CPR, when a person's heart stops beating or a person stops breathing. Doctors press on the chest to help pump blood, and use artificial breathing. Artificial breathing means the doctor puts a tube in the windpipe. Then, a machine breathes for the patient through the tube. Patients usually get medicines by vein. Patients often need an electrical shock to help restart the heartbeat. Without CPR, the heart will not start beating again, and the patient will die.

**3. Artificial Feeding and Fluids**

Doctors use artificial feeding and fluids when people are unable to take enough food and water to stay alive. The food goes through a feeding tube. Usually, the feeding tube goes through the skin into the stomach. Without this treatment, patients die within 710 days.

### Measures: Carers

We used four instruments:

1. Zarit Burden Interview (ZBI) [28]. Comprising 22 items, the ZBI assesses the current burden experienced by caregivers. Items are scored on a rating scale ranging from 0 (never) to 4 (almost always); total scores range between 0 and 88, higher scores indicating greater burden.
2. The Kessler Psychological Distress Scale (K10) [29] is a 10-item measure of psychological distress based on expressed levels of anxiety and depressive symptoms. Items are scored on a rating scale ranging from 1 (none of the time) to 5 (all of the time) with higher scores indicating greater psychological distress.
3. Quality of Carer Patient Relationship (QCPR) [27]. (The PWD had indicated their responses earlier in the interview).
4. The modified LSPQ [24] where the carer was asked to predict those preferences previously made by the person with dementia.

### Statistical analysis

Descriptive statistics were used to summarise the demographic features of the cohort. We report the frequencies and percentages of the ZBI and K10. The scores were also categorised to indicate levels of carer burden e.g. ‘severe carer burden’ (score = 61–88) (ZBI) [30], and ‘moderate mental disorder’ (score = 25–29) (K10). The QCPR scores were dichotomised into ‘good’ and ‘poor’ relationships using the median carer scores, a method developed by Spruttye et al [27]. The median score in the present study cohort was 60, so a dyad was counted as being in the ‘good’ relationship category if either the PWD or the carer achieved a score of 60 or above.

For the LSPQ, we created indices, dichotomizing the scale by collapsing ‘definitely want’, ‘probably want’, and ‘unsure’ responses into a want treatment category, and ‘definitely don't want’ and ‘probably don't want’ into a don't want treatment category [31,32]. We categorized ‘unsure’ responses with ‘want treatment’ responses because the clinical default is often to provide treatment unless specifically refused personally or in an advance decision to refuse treatment. Thus, we assigned a value of zero for ‘do not want treatment’ and one for ‘want treatment’. However, to ensure any level of uncertainty was captured, ‘unsure’ responses were also examined and analysed separately.

The three LSPQ scenarios were expressed in 2x2 tables and described using percentage agreement and the kappa coefficient. Due to the unbalanced distribution of the cell counts in the 2x2 tables the Prevalence Adjusted Bias Adjusted Kappa (PABAK) measure was also applied to all calculations [33]. The level of agreement was described using indices developed by Fleiss et al. [34].

We used the modified LSPQ data to develop an ‘agreement index’ that reflected levels of agreement on treatment preferences. Where a carer could accurately estimate the PWD’s treatment preference, whether that was for active treatment or not, they gained a score of one. Uncertainty only achieved a score of one if that also predicted uncertainty for the treatment preference of the PWD. Thus, we used a score of 0–9; 0 = no ability to predict through to 9 = full ability to predict treatment preferences. Combining scores from all carers gave an overall indication of level of agreement in the sample. Given there was very little missing data for items in measures such as the K10 and ZBI, missing values were replaced by the mean for patients with complete data [35].

Finally, we used multiple linear regression to explore the association between carer and PWD’s characteristics and agreement scores.

### Ethical considerations

The study was approved by the NHS Health Research Authority, NRES Committee South East Coast—Surrey in January 2012 (12/LO/0106).

## Results

### Description of sample

The cohort comprised 60 dyads of a PWD and a family carer or friend recruited from four study sites, the majority recruited from site 1 (see Table 1). Overall we approached 79 dyads over the four sites, of whom 17 declined to participate (76% response rate). The commonest reason for refusal (n = 8) was the carer declining on behalf of the dyad due to their own level of ‘stress’. Demographic characteristics of the cohort are presented in Table 2.

**Table 1:** Recruitment to study.

Site	Dyads who were approached	Dyads who refused or were excluded	Dyads who were interviewed
1. BEH	52	10 (19%)	42 (81%)
2. CPT	9	1 (11%	8 (89%)
3. WLMHT	12	4 (33%)	8 (67%)
4. LPT	6	4 (67%)	2 (33%)
Total	79	19 (24%)	60

Note: Dyad = person with dementia and their family carer/friend; BEH = Barnet Enfield & Haringey MH NHS Trust; CPT = Cambridge and Peterborough Foundation Trust; WLMHT = West London Mental Health Trust; LPT = Leicestershire Partnership MH Trust.

**Table 2:** Characteristics of dyads (n = 60).

	PWD (n = 60)	Carers (n = 60)
**Age** (mean (SD) [range])	79.2 (6.8) [58–93]	66.6 (12.8) [39–93]
**Gender** (% male)	26 (43%)	19 (32%)
**MMSE** (mean (SD) [range])	25.4 (2.4) [20–29]	
**Diagnosis**		
F00.1 (Alzheimer’s late onset)	40 (67%)	
F00.2 (atypical or mixed type Alzheimer’s)	12 (20%)	
other	8 (13%)	
**Ethnicity**		
White British	38 (63%)	42 (70%)
White other*	16 (27%)	12 (20%)
Other**	6 (10%)	6 (10%)
**Previous education**		
Left school ≤ 14 years	14 (23%)	5 (8%)
Left school ≥ 15 years	46 (77%)	55 (92%)
**Living situation of PWD**		
Alone	14 (23%)	
Spouse/partner	37 (62%)	
Other	9 (15%)	
**Relationship to PWD**		
Spouse		35 (58%)
Adult child		18 (30%)
Other		7 (12%)
**Employment—SOC 2010**		
1. Higher managerial, administrative and professional qualifications	28 (47%)	31 (52%)
2. Intermediate occupations	15 (25%)	18 (30%)
3. Routine and manual occupations	16 (27%)	9 (15%)
Never worked or long-term unemployed	0	1
Missing	1	1

Note: PWD = Person with dementia. MMSE = Mini Mental State Examination. ICD 10 = International Disease Classification. SOC = National Statistics Standard Occupational Classification.

* White other includes Irish, Jewish, Greek Cypriot, Turkish Cypriot, Polish and Russian.

** African Caribbean, Indian and Bangladeshi.

### Treatment choices and carer agreement in prediction

LSPQ data are presented as the treatment preferences for active or non-active treatment (see Tables 3 & 4) and the carer’s ability to discern these.

**Table 3:** PWD preference for active treatment (N/%).

Treatment	Scenario 1: As you are today	Scenario 2: Severe stroke with coma	Scenario 3: Advanced cancer
**Antibiotics**	59 (98%)	28 (47%)	28 (47%)
**CPR**	53 (88%)	20 (33%)	18 (30%)
**Tube feeding**	39 (65%)	23 (38%)	22 (37%)

Note: No missing data

**Table 4:** Carer predictions for PWD choice for active treatment (N/%).

Treatment	Scenario 1: As you are today	Scenario 2: Severe stroke with coma	Scenario 3: Advanced cancer
**Antibiotics**	56 (95%)	17 (29%)	33 (56%)
**CPR**	49 (83%)	10 (17%)	19 (32%)
**Tube feeding**	30 (51%)	16 (27%)	22 (37%)

#### Scenario One (‘as you are today’)

Most PWD (98%) expressed a preference to receive antibiotics. However, the preference for active treatment was lower for CPR (88%) and tube feeding (65%). Carer and PWD agreement was highest for antibiotic treatment in scenario one The level of agreement for tube feeding was no better than chance, 20% (***k*** = -0.02; PABAK = -0.60) (see Table 5).

**Table 5:** LSPQ—carer’s ability to accurately estimate the treatment preferences of PWD presented as agreement.

LSPQ Scenario	% Agreement	% Expected agreement	Kappa (*k*)	PABAK
**Scenario 1 ‘As you are today’**				
Antibiotics	71.2	59.4	0.34	0.42
CPR	62.7	45.2	0.30	0.30
Tube feeding	20.3	22.0	-0.018	-0.60
**Scenario 2 ‘Severe stroke with coma’**				
Antibiotics	22.0	24.0	-0.022	-0.60
CPR	42.4	30.0	0.21	-0.20
Tube feeding	44.1	26.0	0.25	-0.12
**Scenario 3 ‘Advanced cancer’**				
Antibiotics	24.0	21.4	0.03	-0.52
CPR	27.1	32.0	-0.07	-0.45
Tube feeding	39.0	23.4	0.20	-0.22

Note: LSPQ = Life Support Preferences Questionnaire; PWD = Person with dementia; PABAK = Prevalence And Bias Adjusted Kappa; CPR = cardio pulmonary resuscitation.

#### Scenario Two (severe stroke with coma)

about half of the PWD showed a preference for no active treatment (antibiotics, 50%; CPR, 57%; tube feeding, 50%) (See Table 3). Carers agreed less in this scenario tending to overestimate the PWD’s desire for no treatment (antibiotic, 67%; CPR, 73%, tube feeding, 66%) (See Table 4). Carers’ ability to estimate the treatment choices of the PWD was lower than in scenario one. In the choice of antibiotic treatment, the strength of agreement was ‘poor’ at 22% (***k*** = -0.022; PABAK = -0.60). There was a 42% level of agreement in the choice of CPR (***k*** = 0.20; PABAK = -0.20). Carers were able to predict the treatment choice of tube feeding with a 44% agreement (***k*** = 0.25; PABAK = -0.12) (See Table 5).

#### Scenario Three (Advanced cancer)

People with dementia were more in favour of antibiotic treatment (47%) than other treatment options (CPR, 30%; tube feeding, 37%) (See Table 3). Carers had similar views to PWD overall (antibiotics, 55%, CPR, 31%; tube feeding, 37%) (See Table 4). However, when it came to concordance within dyads, the level of agreement was low; antibiotic treatment was only 24% which was rated poor (***k*** = -0.03; PABAK = -0.52). For CPR, agreement was also low at 27% but with only ‘poor’ reliability indicated (***k*** = -0.07; PABAK = -0.45). Concordance between the PWD and carer for tube feeding was 39%, however this was only ‘low’ strength (***k*** = 0.20; PABAK = -0.22) (see Table 5).

Antibiotic treatment achieved the highest level of all treatment options in all scenarios at 71% (***k*** = 0.03; PABAK = 0.4).

### Uncertainty

When either the PWD or the carer expressed uncertainty by choosing the ‘unsure’ response on the modified LSPQ, this was potentially lost in the way the modified LSPQ scores were later dichotomised and analysed. In scenario one, PWD were confident in making their preferred treatment choices, and carers agreed with this. However, the selection of the ‘unsure’ response increased for treatments of CPR and tube feeding and within scenarios two and three (see Tables 6 & 7).

**Table 6:** PWD—‘Unsure’ of preference for treatment choice.

Treatment	Scenario 1: As you are today	Scenario 2: Severe stroke with coma	Scenario 3: Advanced cancer	Total
**Antibiotics**	0	2 (3%)	7 (12%)	9 (15%)
**CPR**	1 (1%)	6 (10%)	6 (10%)	13 (22%)
**Tube feeding**	8 (13%)	7 (12%)	7 (12%)	22 (37%)
**Total**	9 (15%)	15 (25%)	23 (35%)	

**Table 7:** Carer–‘Unsure’ of PWD’s preference for treatment choice.

Treatment	Scenario 1: As you are today	Scenario 2: Severe stroke with coma	Scenario 3: Advanced cancer	Total
**Antibiotics**	0	2 (3%)	6 (10%)	8 (14%)
**CPR**	2 (3%)	5 (8%)	4 (7%)	11 (19%)
**Tube feeding**	10 (16%)	3 (5%)	3 (5%)	16 (27%)
**Total**	12 (20%)	10 (17%)	13 (22%)	

### Carer burden, distress and quality of relationship

On the ZBI, almost half of carers (40%) perceived ‘mild to moderate’ levels of carer burden’, 32% ‘little or no burden’ and 27% ‘moderate to severe burden’. Analysis of the K10 indicated the majority of carers fell within the range defined as ‘well’ (score <20; 66.7%). Eight (13.3%) were defined as having a ‘mild mental disorder’, 10% as having a ‘moderate mental disorder’, and 10% a ‘severe mental disorder’ (score ≥ 30). Analysis of QCPR data showed no statistically significant association between the quality of the relationship as perceived by the PWD and that perceived by carers (Pearson’s r = -0.048; P = 0.718).

### Association between carer measures and life support preferences

No significant association was revealed between the Life Support Preference Questionnaire and the variables examined (i.e. care burden, distress and quality of care giver/care receiver relationship).

## Discussion

The treatment preferences of PWD varied across the three scenarios as did family carers’ agreement with those preferences, with a significant degree of decisional uncertainty in both. Perception of relationship quality, carer burden and distress had no effect on carers’ accuracy in estimating the PWD’s treatment preferences.

### Treatment preferences of PWD

People with dementia indicated their preference for life saving treatments in the here and now. The wish to receive life sustaining treatments reduced in the future hypothetical scenarios, especially for CPR and tube feeding.

The hypothetical scenario method can be used to derive information by asking study participants how they would act under certain circumstances [36,37]. Hypothetical scenarios are commonly used in studying sensitive healthcare issues that are difficult to research directly, for example, asking opinions at the bedside of a dying patient [38]. Whilst all the PWD in our study met the essential criteria regarded as necessary to be able to undertake ACP [39,40] and had the capacity to participate, the complex judgments required to place themselves in hypothetical health states may have presented too great a challenge. Our nominal group study [41] showed that PWD find it difficult to contemplate their future selves in hypothetical scenarios, for example, what circumstances might present in the future that may prevent them continuing to live in their own home. This suggests that they would have difficulties making care planning decisions for the future and thus they (as well as their clinicians) may rely on their families and friends. Unfortunately, our results indicate that such reliance may be misplaced.

### Treatment preferences in relation to different health states

There has been little exploration of the future treatment preferences of PWD. This presumably reflects either an assumption that they lack capacity to express a consistent view or else a reluctance to raise sensitive topics with PWD. However, we found that PWD could express their preferences and that there were notable differences across the three health state scenarios. They were more likely to show a preference for antibiotic treatment across all proposed health states but much less likely to opt for more ‘invasive’ treatments, such as tube feeding, this especially so in stroke with coma and this concurs with the findings of Low et al.[42] in interviews with nursing home residents who did not have dementia

Our findings are consistent with other studies investigating the views of older people [43,44] where participants (who did not have dementia) wished to forego treatments in the event of being in a coma. However, Scandrett et al. [45] found little consistency in treatment preferences across a range of hypothetical health states. However, neither of these studies involved participants with dementia; indeed, dementia was presented as one of the hypothetical scenarios.

We cannot assume that in the event of a diagnosis of dementia that people’s end of life care treatment and care preferences will change. Indeed, our findings suggest that they maybe do not change much. Therefore, any discussions about end of life care should start with the PWD. Schellinger et al. [46] advocated the use of disease-specific advance care planning, conducted by clinicians who are knowledgeable and skilled in communication relating to the life limiting condition with which a person is diagnosed. Though they did not test this approach in dementia, Schellinger and colleagues reported improvements in end of life care communication for people with heart disease, with a correspondingly increased access to hospice care.

### Carers’ agreement with the treatment preferences of PWD

Carers’ agreement with the end of life treatment preferences of the PWD varied across the three scenarios. Most carers agreed with the PWD’s preferences in the here and now, perhaps because it is a *known* situation that requires less consideration, concurring with findings of similar studies [47]. However, as the scenarios increasingly focused on severe future illness states, carers’ and PWD’s level of agreement became, at best, moderate. The more interventional treatments, such as CPR and tube feeding, showed lower levels of agreement across all scenarios. This may be due in part to most carers having previous knowledge and experience of antibiotic treatment whereas CPR and tube feeding may be less familiar to them or indeed be seen as more emotive and ‘high stakes’ as treatment options. Overall, carers showed a moderate to low ability to estimate the treatment preferences of the PWD, and lower than that found in other research [13,41,47].

In general, research on carers’ ability to predict accurately the preferences of a PWD have produced conflicting findings. Whitlach et al [12] examined the accuracy of family carers’ ability to estimate the PWD’s treatment preferences and concluded it to be ‘adequate’, but without specifying what ‘adequate’ actually meant. In contrast, Shalowitz et al [47] found that, in one-third of cases, next-of-kin and patient-nominated decision makers incorrectly predicted patients' end of life treatment preferences. In their review, they concluded that even discussing preferences for treatment or designating a person to make decisions on their behalf failed to improve the surrogates' predictive accuracy.

### Factors that might influence accuracy

Our study revealed no association between carer burden and distress or quality of relationship that influences the ability to predict accuracy in decision making, which is not consistent with other studies [12,48]. Carers may find it difficult to reconcile their own emotional needs with those of the person they care for, struggling to decide what they think the person would have wanted [49]. Although our study did not incorporate a specific measure for this, we found that both PWD and carers showed marked uncertainty for treatment choices in future scenarios and choosing the ‘unsure’ response in the LSPQ scale. This is common in dementia care around issues such as imprecision in diagnosis, lack of information and communication about prognosis [50] and lack of knowledge about care and treatment options for the future [51]. Mishel [52] proposed a ‘theory of uncertainty’ whereby, especially in chronic illness-related situations, the decision maker finds it very stressful and difficult to make a judgment or to predict what an individual might or might not want due to lack of cues and information [53]. In an earlier study [41] we found that family carers perceived a future with dementia as bleak, which may in part be due a lack of support, communication and information on what the future holds for them as a carer, but this may also influence the decisions they think the PWD would make for themselves. However, the preferences expressed by the PWD in the earlier stages of the disease may not be consistent as they move through the illness and adapt to the diagnosis; their views on what constitutes a good quality of life may change [54].

### Methodology and limitations of the study

This study is the first to have used the LSPQ with PWD in the UK in considering treatment options in hypothetical illness scenarios. The research was undertaken by one interviewer on a sample that may not be representative of this population. However, issues such as the inevitable worsening of dementia, impending loss of decision-making capacity, and the likelihood of future physical illness are universal for all people affected by this condition and their families and friends.

Recruitment to the study was challenging, despite being extended across four geographical areas and two dementia research registers. Requiring consent of both parties of the dyad may have been restrictive in recruitment with either the PWD or their carer in any potential dyad refusing to take part. As in other studies, carers tended to ‘gate keep’ researcher access to the PWD [55,56]. Some clinicians were cautious in recommending some dyads, reasoning that they had only recently received the diagnosis and it was too soon to seek their interest in a study on ACP and end of life care. This caution may reflect clinician reluctance to discuss the nature of the research topic for fear of causing distress; however no potential participants expressed any distress or concern regarding the topic.

A PWD may find end of life care issues especially difficult to process. Whilst approaches, such as the LSPQ, may attempt to mimic actual healthcare scenarios, they are in danger of being minimalist in their descriptions, lacking specific detail and failing to reflect how complex and emotionally draining decision making can be. This may lead participants to make choices that may not reflect the reality of a future health state or indeed reflect their wishes and preferences in the event, so we cannot assume that participants would make these choices when faced with such a situation. Nor do we know if the PWD’s wishes are simply overridden by the carer if those views were different. However, the actual outcome of events for these dyads is not what we measured here. Rather, of interest was carer agreement and the extent to which different scenarios led to different patterns of agreement.

The lack of association between measures of the carer relationship, carer burden and levels of agreement on health preferences was not entirely expected. However, we must emphasise that this study was may not have been powered to examine these associations.

### Significance of this research

This study contributes novel findings about decision making and how PWD and carers might make choices about future health care. A key finding of this study was that carer burden and distress did not seem to influence a carer’s ability to estimate the preferences of his or her PWD, therefore further exploration is required to understand in more depth the complexities of proxy decision making for PWD at end of life.

### Clinical implications

The work has clinical implications, for example further demonstrating the need to take time to ensure that the PWD (especially) have the relevant information to make their own choices, where possible, and that these are clearly communicated and recorded, so that any ACP is readily available when required in the future.

There is often an assumption in practice [13,57] that carers and PWD will speak with one voice but we found this cannot be assumed. This suggests that there is a need for targeted educational initiatives and support, on the nature and course of dementia but also on how to plan ahead and with that dialogue to be revisited and reviewed at frequent intervals. We will need to give more consideration to the problem of uncertainty if we are to support families in decision making as the dementia progresses and also to prepare professionals [58] in providing appropriate support.

### Conclusion

Whilst robust evidence for the presumed benefits of end of life communication and ACP in dementia is still lacking, this study furthers our understanding of the challenges that face PWD and their families in considering end of life care treatment preferences. Advance care planning may offer a range of benefits to PWD and their families such as initiating conversations that lead to thinking ahead and articulating wishes and preferences for care in the future. But, there remain significant barriers that will need to be addressed in order to gain optimum outcomes of any such intervention. PWD find it difficult to conceive of their future selves and think about preferences for end of life care and in the absence of a process of continued communication this makes it difficult for them to identify future treatment choices for any possible hypothetical health scenario they may experience. Moreover, their carers, who may be forced to make proxy decisions, are often not aware of, or cannot estimate, their preferences.

## Abbreviations

CPR: Cardio-pulmonary resuscitation
K10: Kessler Psychological Distress Scale
LSPQ: Life Support preferences Questionnaire
MMSE: Mini Mental State Examination
PABAK: Prevalence-Adjusted Bias-Adjusted Kappa
PWD: People with dementia
QCPR: Quality of Carer Patient Relationship
ZBI: Zarit Burden Inventory
