# Correction: Advance Care Planning in Dementia: Do Family Carers Know the Treatment Preferences of People with Early Dementia?

**DOI:** 10.1371/journal.pone.0161142

**Published:** 2016-08-09

**Authors:** Karen Harrison Dening, Michael King, Louise Jones, Victoria Vickerstaff, Elizabeth L. Sampson

The fourth author’s name is spelled incorrectly. The correct name is: Victoria Vickerstaff.
